# Multiple Myeloma Presenting as a Pancoast-Like Tumor: A Diagnostic Challenge

**DOI:** 10.7759/cureus.117093

**Published:** 2026-09-28

**Authors:** Maria João Lúcio, Cristina Matos, Lucília Carvalho, Tiago Saldanha, José M Correia

**Affiliations:** 1 Pulmonology, Centro Hospitalar de Lisboa Ocidental, Lisbon, PRT; 2 Pathology and Laboratory Medicine, Centro Hospitalar de Lisboa Ocidental, Lisbon, PRT; 3 Radiology, Centro Hospitalar de Lisboa Ocidental, Lisbon, PRT

**Keywords:** art of diagnosis, diagnosis of multiple myeloma, extra-medullary plasmacytoma, pancoast tumor, pancoast tumour, thoracic oncology

## Abstract

Apical lung masses are classically attributed to bronchogenic carcinoma, particularly superior sulcus (Pancoast) tumors. However, other entities may mimic this presentation. We report the case of a 68-year-old man, a former smoker, referred for progressive fatigue and lower limb edema, with normocytic anemia and an elevated erythrocyte sedimentation rate. Thoracoabdominal computed tomography revealed a 4.5-cm solid mass in the right lung apex with rib destruction and T2 vertebral body involvement, suggestive of a Pancoast tumor. Positron emission tomography demonstrated intense fluorodeoxyglucose (FDG) uptake (maximum standardized uptake value (SUV_max_) 11.3) and a hypermetabolic osteolytic lesion at T10. CT-guided transthoracic biopsy revealed infiltration by atypical plasma cells, CD138-positive and thyroid transcription factor 1 (TTF-1)-negative, with lambda light chain restriction. Bone marrow evaluation confirmed multiple myeloma. This case underscores the importance of histopathological confirmation in apical masses and the need to include multiple myeloma in the differential diagnosis, particularly when systemic hematologic abnormalities are present.

## Introduction

Superior pulmonary sulcus tumors are most commonly associated with non-small cell lung cancer (NSCLC) and may invade adjacent ribs, vertebrae, and neural structures. Invasion of the thoracic inlet may cause Pancoast syndrome, characterized by shoulder or arm pain in the C8-T2 distribution, intrinsic hand-muscle weakness or atrophy, and/or Horner syndrome [1-4].

However, the thoracic apex represents a complex anatomical region where a variety of neoplastic and non-neoplastic conditions may arise, including lymphomas, metastatic disease, infections, and, less commonly, plasma cell dyscrasias, and sometimes can mimic primary lung cancer [5,6]. 

Multiple myeloma (MM) is a clonal plasma cell neoplasm commonly associated with hypercalcemia, renal dysfunction, anemia, and osteolytic bone lesions (CRAB features). An osteolytic lesion may breach the cortex and extend into adjacent soft tissue, forming a paraskeletal (bone-related) plasmacytoma. This differs from true extramedullary disease, which arises in soft tissue without direct continuity with bone. Presentation as an apical paraskeletal mass is uncommon and may pose a diagnostic challenge [3,7,8].

We report a case of MM presenting as a Pancoast-like tumor, highlighting the importance of histopathological confirmation and a broad differential diagnosis in patients with apical masses.

## Case presentation

A 68-year-old man, a former smoker (cessation 40 years prior), with a medical history of arterial hypertension, dyslipidemia, and pacemaker implantation for cardiac arrhythmia, was referred for evaluation of progressive fatigue and bilateral lower limb edema. There was no family history of lung cancer. His occupational history was notable for prolonged exposure to chemical agents in the automobile industry. Symptoms had an insidious onset and were later accompanied by productive cough, pain, and paresthesia of the lower limbs. He denied dyspnea or chest pain. A brief neurological examination revealed no abnormalities. Shoulder or arm pain, upper-limb neurological symptoms, and signs of Horner syndrome were not documented.

Laboratory evaluation revealed normocytic, hypochromic anemia, with a hemoglobin level of 10.9 g/dL (reference range: 13.0-17.0 g/dL), low serum iron, and a transferrin saturation of 20% (reference range: 20-50%). Inflammatory markers were elevated, with an erythrocyte sedimentation rate of 98 mm/h (reference value: <20 mm/h) and a C-reactive protein level of 6 mg/dL (reference value: <0.50 mg/dL).

Computed tomography (CT) of the chest, abdomen, and pelvis (Figure 1) identified a 4.5-cm solid right apical mass with destruction of adjacent ribs and involvement of the T2 vertebral body, raising suspicion for a primary lung malignancy involving the superior pulmonary sulcus. No mediastinal or hilar lymphadenopathy or pleural or pericardial effusion was observed. Additional findings included bilateral adrenal nodules and small para-aortic lymph nodes of uncertain significance. No dedicated spinal imaging was performed; therefore, neural foraminal, epidural, or spinal canal extension could not be reliably excluded. 

**Figure 1:**
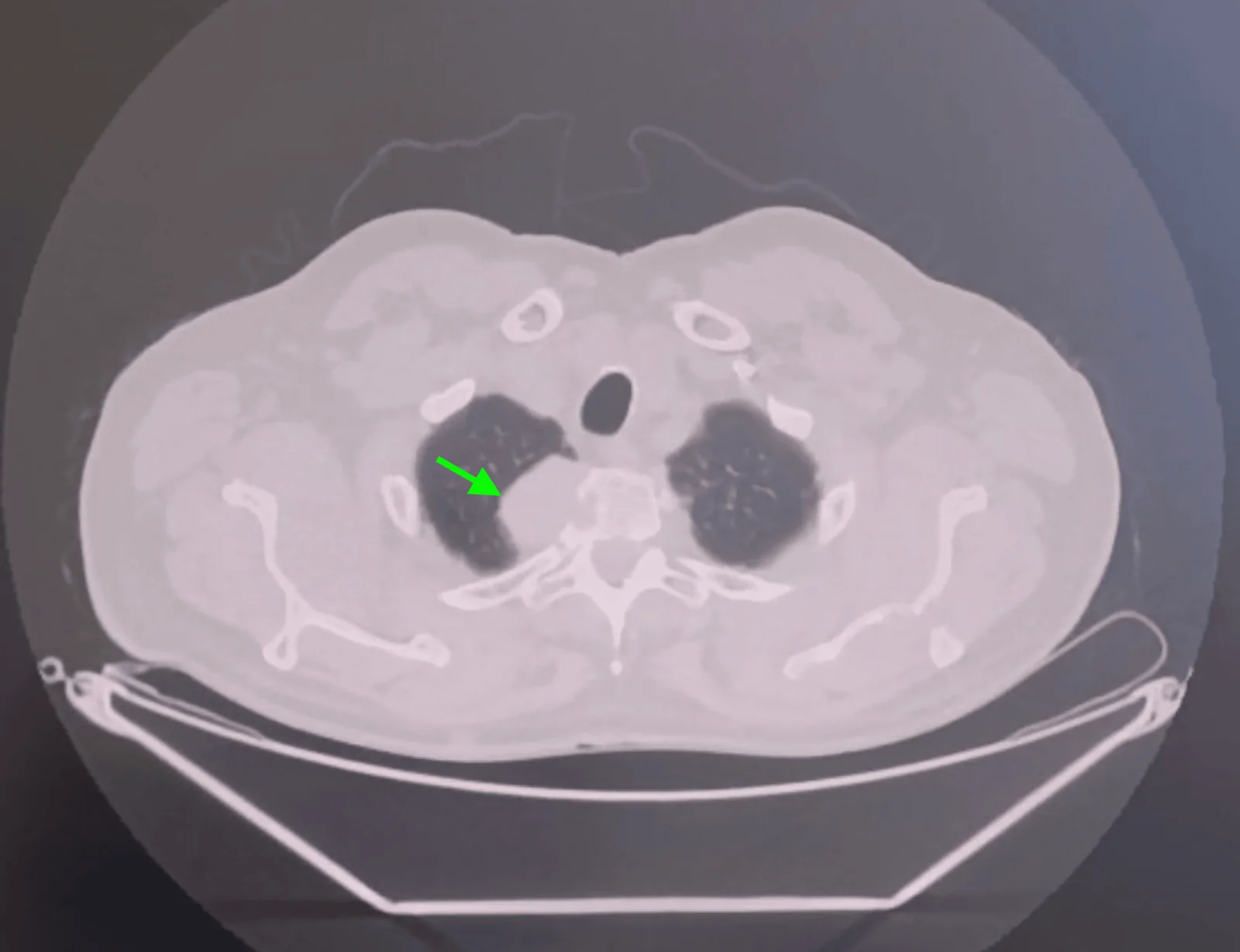
Chest computed tomography Chest CT with a 4.5 cm solid mass in the right lung apex. The green arrow points to the lung mass.

Positron emission tomography/computed tomography (PET/CT) demonstrated intense FDG uptake in the right apical mass (maximum standardized uptake value (SUV_max_) 11.3) (Figure 2), with extension into the chest wall and involvement of the adjacent vertebral body. A separate hypermetabolic osteolytic lesion was identified at T10 (SUV_max_ 6.7), initially raising concern for distant metastasis in the setting of suspected lung cancer. A less well-defined acetabular lesion was also considered suspicious. The bilateral adrenal nodules and small para-aortic lymph nodes seen on the initial CT showed no suspicious fluorodeoxyglucose (FDG) uptake on PET/CT.

**Figure 2:**
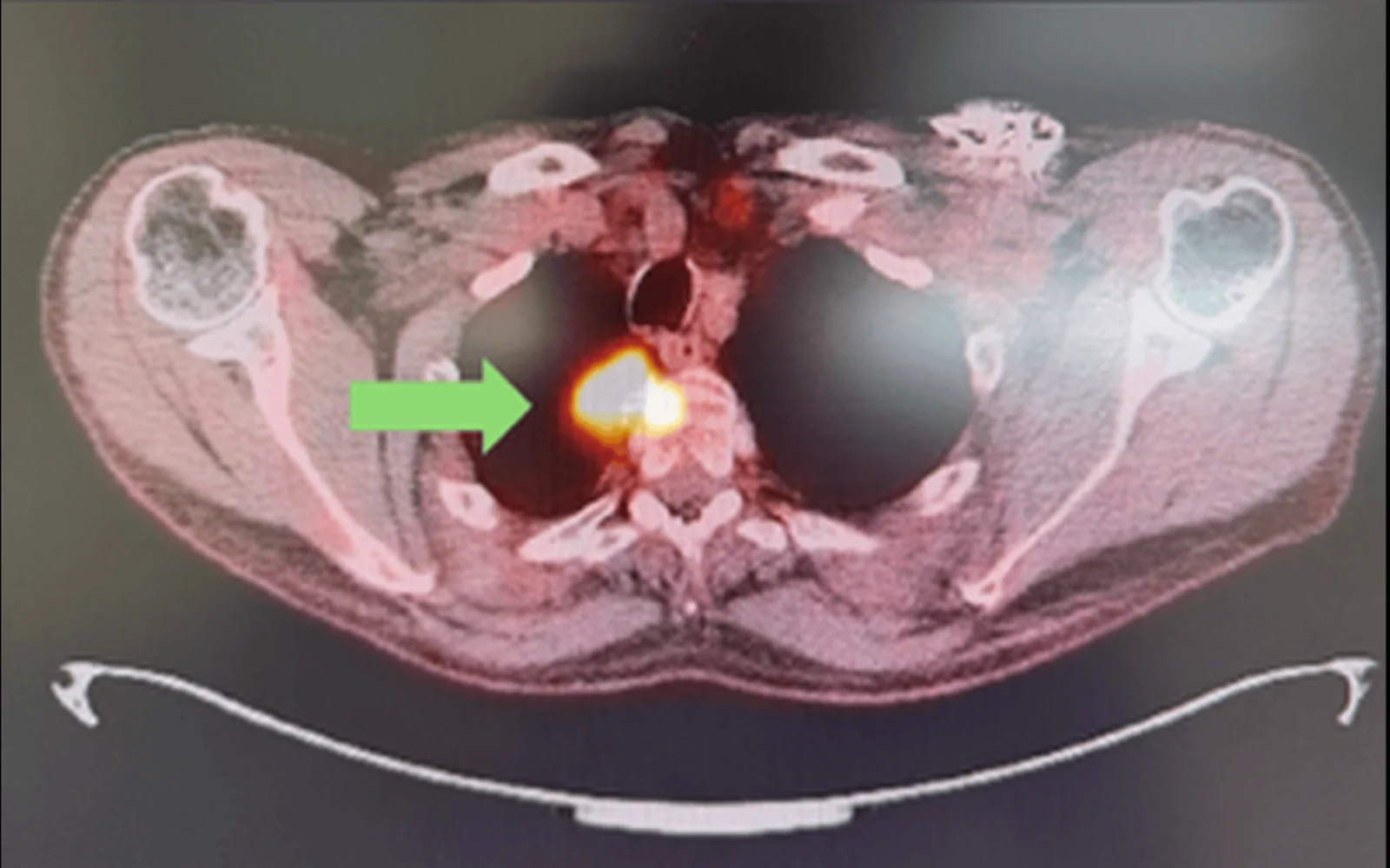
FDG-avid right apical mass on PET/CT Consecutive axial fused ¹⁸F-FDG PET/CT images show intense uptake in the right apical mass (green arrows; SUV_max_ 11.3), with adjacent chest wall and vertebral body involvement. FDG: fluorodeoxyglucose; PET/CT: positron emission tomography/computed tomography; SUV_max_: maximum standardized uptake value.

Given the imaging findings, primary lung malignancy was initially considered. However, CT-guided transthoracic biopsy revealed diffuse infiltration by atypical plasma cells. Immunohistochemistry was positive for CD138 and negative for TTF-1, and in situ hybridization demonstrated lambda light-chain restriction, consistent with a plasma cell neoplasm (Figure 3). The patient was subsequently referred to hematology, where bone marrow evaluation revealed 10-12% clonal plasma cells. This finding, together with the osteolytic lesions at T2 and T10 interpreted as myeloma-related bone involvement, supported the diagnosis of multiple myeloma rather than solitary plasmacytoma.

**Figure 3:**
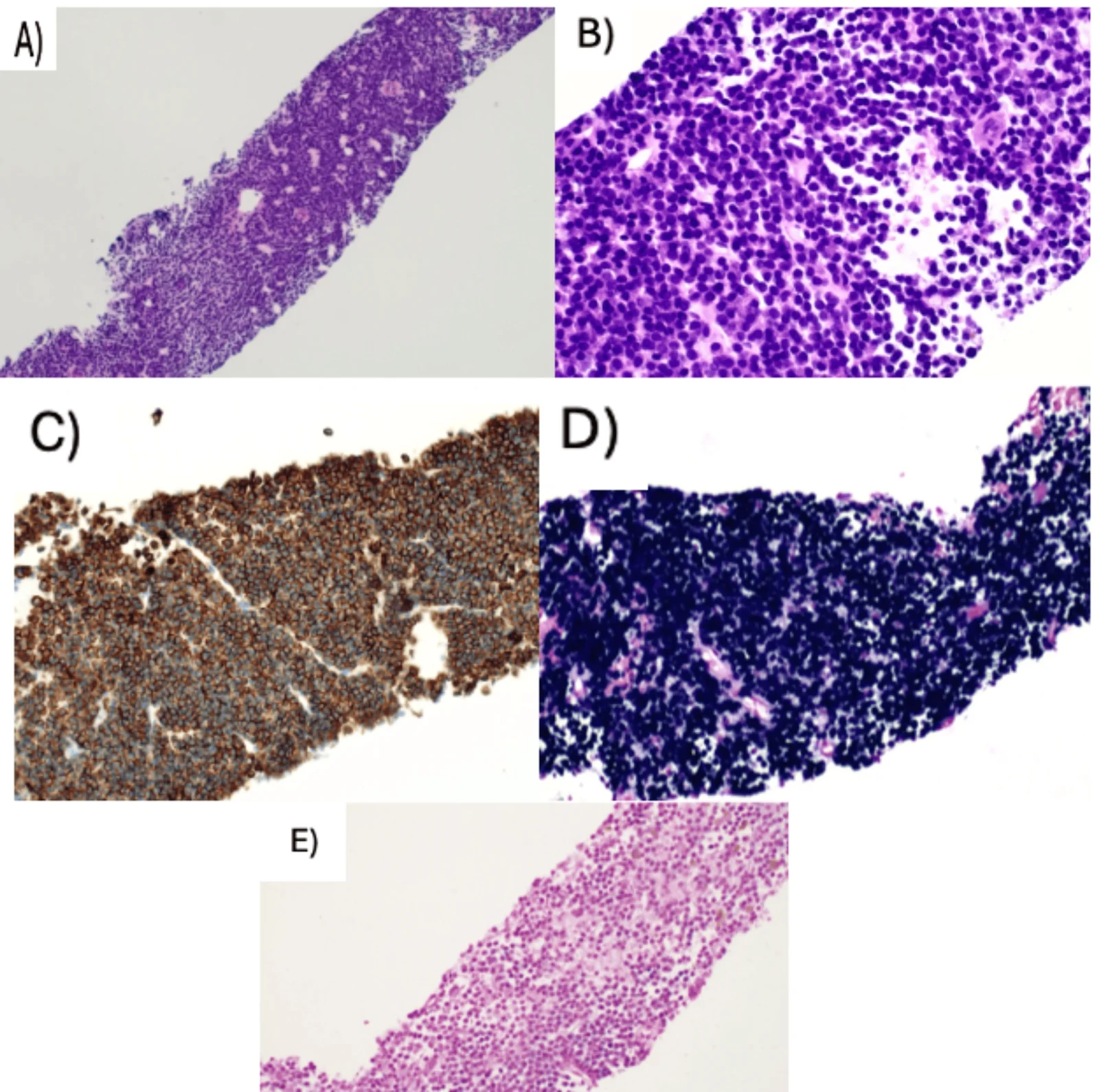
Histopathological findings of the CT-guided transthoracic biopsy (A) Low-power hematoxylin and eosin staining showing diffuse infiltration of the biopsy core by monomorphic plasmacytoid cells (×40). (B) High-power view demonstrating atypical plasma cells with plasmacytoid morphology (×400). (C) Immunohistochemistry showing diffuse CD138 expression (×20). (D-E) In situ hybridization for immunoglobulin light chains showing absent or minimal kappa expression (D) and diffuse lambda expression (E), confirming lambda light-chain restriction (×20).

## Discussion

Apical thoracic masses with invasion of adjacent ribs, vertebrae, or neural structures may raise suspicion for primary lung malignancy, particularly NSCLC. In this setting, a solid apical lesion with bone destruction and intense FDG uptake, as in our patient, may suggest primary lung cancer. These imaging findings alone do not establish Pancoast syndrome; the lesion is therefore described as a Pancoast-like superior sulcus mass. However, the thoracic apex is a complex anatomical region where a broad spectrum of diseases may arise, including metastatic disease, primary pulmonary lymphoma, and infectious or inflammatory processes, which can closely mimic primary lung carcinoma on imaging. Plasma cell neoplasms are rarely considered in this differential. MM is defined by ≥10% clonal bone marrow plasma cells or a biopsy-proven bony or extramedullary plasmacytoma, together with at least one myeloma-defining event, comprising attributable CRAB features or validated biomarkers of malignancy. In this patient, 10-12% clonal bone marrow plasma cells and osteolytic lesions attributed to myeloma fulfilled these criteria, with bone disease constituting the myeloma-defining event. Myeloma-associated osteolytic lesions may extend into adjacent soft tissues and resemble solid tumors [1,9].

Myeloma bone disease, present in 80-90% of patients at diagnosis, results from pathological upregulation of osteoclast activity via the receptor activator of nuclear factor kappa B (RANK)/receptor activator of nuclear factor kappa B ligand (RANKL) pathway and suppression of osteoblast function, leading to lytic lesions that may breach cortical bone and extend into adjacent soft tissues. Previous cases of plasmacytoma involving upper thoracic vertebrae or ribs have presented as apical masses or Pancoast-like syndrome, initially interpreted as primary lung cancer until biopsy revealed a plasma cell neoplasm [5,10,11].

Clinically, several features in this patient were atypical for primary lung carcinoma. The patient’s fatigue, normocytic anemia, and markedly elevated erythrocyte sedimentation rate were nonspecific and did not reliably distinguish a hematologic malignancy from primary lung carcinoma. Similarly, the absence of prominent respiratory symptoms and the lack of documented classical Pancoast-like syndrome features did not exclude an apical lung primary. Histopathological assessment was therefore decisive: the combination of atypical plasmacytoid morphology, diffuse CD138 expression, and lambda light-chain restriction supported a plasma cell neoplasm and prompted further hematological evaluation. TTF-1 negativity alone does not exclude primary lung carcinoma, and CD138 expression must be interpreted alongside morphology and the other immunophenotypic findings [12,13].

Our findings align with the limited literature describing plasma cell neoplasms that present as an apical mass or Pancoast-like syndrome, often mistaken for primary lung cancer until biopsy confirms the actual diagnosis. Chen and Padmanabhan [5] documented this for extramedullary plasmacytoma, Goździuk et al. [14] for solitary plasmacytoma, and Sandberg et al. [15] for primary plasma cell leukemia. These reports reinforce the importance of tissue confirmation in evaluating superior sulcus masses, as emphasized by Arcasoy and Jett [1], and illustrate that similar thoracic presentations may occur in distinct plasma cell disorders. In our patient, clonal bone marrow involvement and separate osteolytic lesions supported multiple myeloma. The associated rib destruction and vertebral involvement raise the possibility of a paraskeletal plasmacytoma arising through contiguous soft-tissue extension from an osseous lesion. However, the available imaging information does not establish the anatomical epicenter or direction of spread, preventing a definitive distinction between paraskeletal involvement and true extramedullary disease. The latter refers to hematogenous soft-tissue involvement without direct continuity with bone and is generally associated with poorer outcomes than paraskeletal involvement [16,17]. In our patient, atypical plasmacytoid morphology, diffuse CD138 expression, and lambda light-chain restriction collectively supported a plasma cell neoplasm. This highlights the importance of interpreting morphology and immunophenotypic findings together rather than relying on any single marker [18,19].

This case has limitations inherent to a single-case observation and cannot be generalized. Nevertheless, it reinforces a fundamental principle of thoracic oncology: histopathological confirmation is mandatory before establishing a diagnosis of lung cancer and initiating definitive therapy.

## Conclusions

MM, although uncommon in this location, should be included in the differential diagnosis of apical masses, particularly when multifocal osteolytic lesions and anemia are present. This case highlights the importance of integrating imaging, laboratory findings, and tissue diagnosis: biopsy identified a clonal plasma cell neoplasm, while subsequent bone marrow and skeletal evaluation supported the diagnosis of MM. The superior sulcus mass did not establish a clinical diagnosis of Pancoast syndrome, and the available imaging information could not definitively distinguish between paraskeletal involvement and true extramedullary disease. Recognition of such presentations can help guide appropriate diagnostic evaluation.

## References

[1] Arcasoy SM, Jett JR (1997). Superior pulmonary sulcus tumors and Pancoast's syndrome. N Engl J Med.

[2] Foroulis CN, Zarogoulidis P, Darwiche K (2013). Superior sulcus (Pancoast) tumors: current evidence on diagnosis and radical treatment. J Thorac Dis.

[3] Kozower BD, Larner JM, Detterbeck FC, Jones DR (2013). Special treatment issues in non-small cell lung cancer: diagnosis and management of lung cancer, 3rd ed: American College of Chest Physicians evidence-based clinical practice guidelines. Chest.

[4] Rusch VW (2006). Management of pancoast tumours. Lancet Oncol.

[5] Chen KT, Padmanabhan A (1983). Pancoast syndrome caused by extramedullary plasmacytoma. J Surg Oncol.

[6] White HD, White BA, Boethel C, Arroliga AC (2011). Pancoast's syndrome secondary to infectious etiologies: a not so uncommon occurrence. Am J Med Sci.

[7] Mikhael J, Ismaila N, Cheung MC (2019). Treatment of multiple myeloma: ASCO and CCO joint clinical practice guideline. J Clin Oncol.

[8] van de Donk NW, Pawlyn C, Yong KL (2021). Multiple myeloma. Lancet.

[9] Cowan AJ, Green DJ, Kwok M (2022). Diagnosis and management of multiple myeloma: a review. JAMA.

[10] Mukkamalla SK, Malipeddi D (2021). Myeloma bone disease: a comprehensive review. Int J Mol Sci.

[11] Terpos E, Ntanasis-Stathopoulos I, Gavriatopoulou M, Dimopoulos MA (2018). Pathogenesis of bone disease in multiple myeloma: from bench to bedside. Blood Cancer J.

[12] Marisavljevic D, Markovic O, Cemerkic-Martinovic V, Ponomarev D (2005). Plasmacytoma of the lung: an indolent disease resistant to conventional myeloma treatment: report of a case. Med Oncol.

[13] Shin MS, Carcelen MF, Ho KJ (1992). Diverse roentgenographic manifestations of the rare pulmonary involvement in myeloma. Chest.

[14] Goździuk K, Kedra M, Rybojad P, Sagan D (2009). A rare case of solitary plasmacytoma mimicking a primary lung tumor. Ann Thorac Surg.

[15] Sandberg Y, Verhoeven GT, Weerkamp F, Broyl A, Emmering J, Budel LM (2020). Primary plasma cell leukemia mimicking a Pancoast tumor. Am J Hematol.

[16] Xu X, Wang Y, Zhang W (2026). Prognosis of extramedullary disease in Chinese newly diagnosed multiple myeloma patients treated with novel agents-based induction therapy. Cancer Med.

[17] Bladé J, Beksac M, Caers J (2022). Extramedullary disease in multiple myeloma: a systematic literature review. Blood Cancer J.

[18] Möller K, Gulzar T, Lennartz M (2024). TTF-1 is a highly sensitive but not fully specific marker for pulmonary and thyroidal cancer: a tissue microarray study evaluating more than 17,000 tumors from 152 different tumor entities. Virchows Arch.

[19] O'Connell FP, Pinkus JL, Pinkus GS (2004). CD138 (syndecan-1), a plasma cell marker immunohistochemical profile in hematopoietic and nonhematopoietic neoplasms. Am J Clin Pathol.

